# Periorbitale heliotrope Erytheme mit Ödem als potenzieller klinischer Indikator für eine paraneoplastische Dermatomyositis – eine Fallserie

**DOI:** 10.1111/ddg.70178

**Published:** 2026-06-04

**Authors:** Antigona Aliu, Ulrich Peter Wehry, Frank Oellig, Thilo Gambichler, Sven‐Niklas Burmann, Milan Vidakovic, Julian Kionke, Jörg H. W. Distler, Andrea‐Hermina Györfi, Valentina Laura Müller, Alexander Kreuter

**Affiliations:** ^1^ Klinik für Dermatologie Venerologie und Allergologie Helios St. Elisabeth Krankenhaus Oberhausen Universität Witten/Herdecke; ^2^ Pathologie Mülheim an der Ruhr Mülheim an der Ruhr, Deutschland; ^3^ Klinik für Dermatologie Klinikum Dortmund Universität Witten‐Herdecke Fakultät für Gesundheit/Medizinische Fakultät, Dortmund; ^4^ Klinik für Dermatologie Christliches Klinikum Unna, Unna; ^5^ Klinik für Dermatologie Venerologie und Allergologie Helios St. Johannes Krankenhaus Duisburg; ^6^ Klinik für Rheumatologie und Hiller‐Forschungszentrum Universitätsklinikum Düsseldorf, Düsseldorf

**Keywords:** Dermatomyositis, heliotropes Erythem, Lidödem, Anti‐TIF1‐γ, Anti‐NXP‐2, Paraneoplastisches Syndrom, Dermatomyositis, heliotrope erythema, lid edema, anti‐TIF1‐γ, anti‐NXP‐2, paraneoplastic syndrome

## Abstract

Die Dermatomyositis (DM) im Erwachsenenalter kann eine enge Assoziation mit Malignomen aufweisen, insbesondere bei Nachweis von Anti‐TIF1‐γ‐ oder Anti‐NXP‐2‐Antikörpern. Diese Fallserie beschreibt zehn Fälle von DM mit ausgeprägten periorbitalen heliotropen Erythemen und Ödemen (PHEÖ), in der bei neun Patienten in der apparativen Diagnostik zugrundeliegende Malignome gesichert wurden. Unter Einbeziehung zusätzlicher, in der Literatur beschriebener serologischer Marker und klinischer Charakteristika wird eine einfache Kriterienliste vorgeschlagen, die bei der Beurteilung einer paraneoplastischen Genese bei DM hilfreich sein kann. Das Vorliegen eines PHEÖ scheint ein bedeutsames klinisches Anzeichen für eine paraneoplastische DM zu sein und erfordert eine sorgfältige Malignomabklärung.

## SUMMARY

Adult‐onset dermatomyositis (DM) is frequently associated with underlying malignancies, especially in individuals with anti‐TIF1‐γ or anti‐NXP‐2 autoantibodies. This case series describes 10 cases of DM with pronounced periorbital heliotropic erythema and eyelid edema (PHEÖ), in which an underlying malignancy was confirmed by imaging in 9 patients. By incorporating additional serological markers and clinical characteristics described in the literature, a simple criteria list is provided to aid in the assessment of a paraneoplastic etiology in DM. The presence of PHEÖ appears to constitute a clinically relevant indicator of paraneoplastic DM and requires careful screening for malignancy.

## EINLEITUNG

Die Dermatomyositis (DM) im Erwachsenenalter ist eine entzündliche Myopathie, die in einem relevanten Anteil der Fälle mit Malignomen im Sinne eines paraneoplastischen Syndroms (PNS) assoziiert ist.^1^ Die zugrundeliegenden pathophysiologischen Mechanismen der DM als PNS sind bislang nicht vollständig geklärt. Es wird angenommen, dass Tumorantigene strukturelle Ähnlichkeiten zu Muskelantigenen aufweisen und dadurch im Rahmen einer antitumoralen Immunreaktion eine Myositis auslösen können.^2^ Neben diesem Konzept des *molecular mimicry* wird zudem die aberrante Expression Myositis‐spezifischer Autoantigene im Tumorgewebe diskutiert. Beide Hypothesen könnten erklären, weshalb bei einem Teil der Patienten ein Malignom zugrunde liegt und somit DM als PNS auftreten kann.^3^, ^4^

Bei Erwachsenen wird für etwa 15–30 % der DM‐Fälle eine paraneoplastische Genese angenommen, wobei die Prävalenz je nach Kohorte, Altersgruppe und angewandter Methodik stark variiert.^5^, ^6^, ^7^ Besonders häufig treten in diesem Zusammenhang Adenokarzinome der Ovarien, des Urothels und der Lunge auf, wenngleich auch über diverse andere Tumorentitäten berichtet wird.^8^ Serologisch sind insbesondere Anti‐Transkription‐Intermediär‐Faktor‐1‐γ (TIF1‑γ) und Anti‐Nuclear‐Matrix‐Protein‑2 (NXP‑2)‐Antikörper mit paraneoplastischer DM assoziiert.^9^, ^10^ In einer Kohortenanalyse von über 200 Patienten wiesen mehr als 80 % der Fälle mit malignitätsassoziierter DM Antikörper gegen NXP‐2 (31 %) oder TIF‑1γ (52 %) auf.^11^ In einer retrospektiven Fallsammlung erwachsener Patienten mit DM aus Deutschland betrug der positive prädiktive Wert (PPW) von Anti‑TIF1‑γ‐Antikörpern 100 %, da alle Patienten mit positivem Titer ein Malignom aufwiesen, während Anti‑TIF1‑γ‐negative Patienten kein Malignom entwickelten.^12^ Allerdings zeigen sowohl systematische Übersichtsarbeiten als auch Kohortenanalysen, dass Anti‑TIF1‑γ‐Antikörper nicht obligat auf eine paraneoplastische Genese hinweisen. Jedoch wurde in einer Meta‐Analyse ein etwa 9,4‑fach erhöhtes Malignomrisiko beschrieben.^13^ In einer weiteren Fallserie wiesen lediglich 27 % der Anti‑TIF1‑γ‐positiven Patienten eine Neoplasie auf.^14^ Somit entwickelt ein signifikanter Anteil der Anti‑TIF1‑γ‐positiven Patienten trotz nachweisbarer Autoantikörper kein Malignom.^15^

Assoziierte Neoplasien können sowohl vor als auch (mehrere Jahre) nach der Erstdiagnose der DM auftreten.^1^ Am häufigsten werden jedoch zugrunde liegende Tumorerkrankungen innerhalb des ersten Jahres nach Erstdiagnose einer DM entdeckt. Die Wahrscheinlichkeit, einen assoziierten Tumor bei der DM zu detektieren, nimmt im Laufe der Folgejahre deutlich ab.^5^, ^6^, ^7^ Des Weiteren konnte gezeigt werden, dass Tumor‐Rezidive oder eine Metastasierung eines Tumors eine DM auslösen oder verschlimmern können.^10^, ^16^ Die frühzeitige Detektion bisher unerkannter Neoplasien stellt somit einen entscheidenden prognostischen Faktor dar. Zu den charakteristischen kutanen Phänomenen der DM zählen, neben den „Gottron Papeln“ an den Fingern, auch konfluierende Erytheme am Oberkörper (*Shawl sign*) und im Gesicht (heliotropes Erythem). In einer retrospektiven Untersuchung aus dem Jahr 2023 erwies sich das heliotrope Erythem bei DM als unabhängiger Risikofaktor für Tumorassoziation, insbesondere bei Krankheitsbeginn nach dem 40. Lebensjahr.^17^ Zudem wurden weitere Faktoren (kutane Nekrosen, Dysphagie, männliches Geschlecht oder rapider Beginn der Myositis) identifiziert, die mit einer paraneoplastischen DM assoziiert sind.^18^ Die vorliegende Fallserie beschreibt Patienten mit heliotropen Erythem und Ödem (PHEÖ) als klinisches Anzeichen für das Vorliegen einer paraneoplastischen DM.

## PATIENTENKOLLEKTIV

**ABBILDUNG 1:**
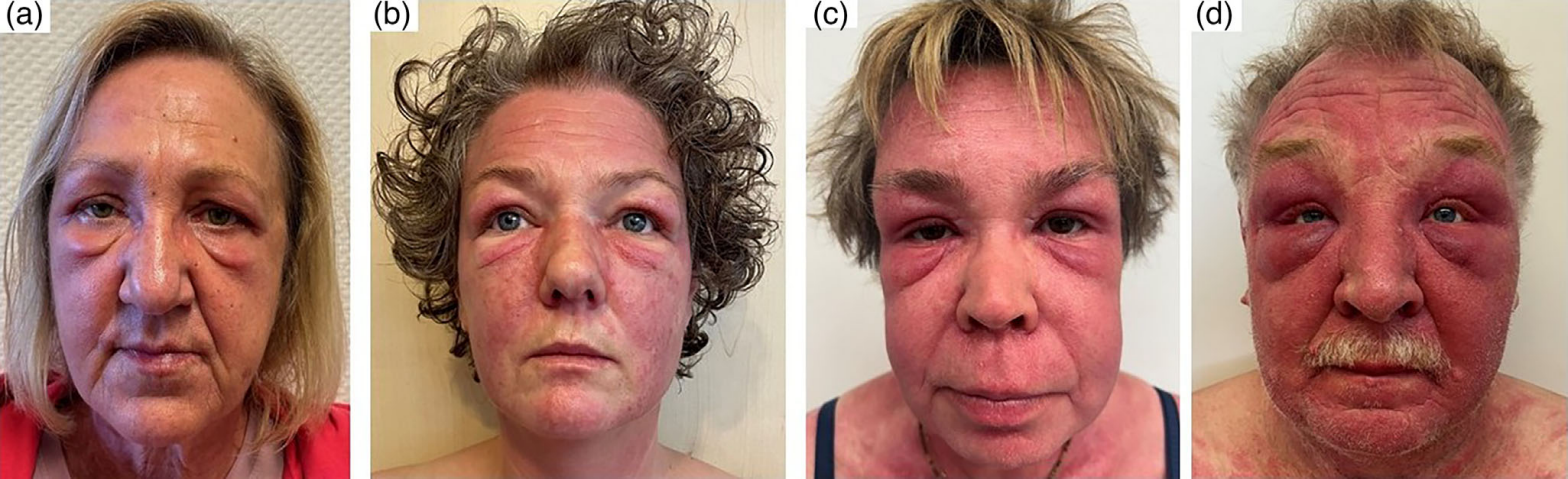
Klinisches Spektrum der periorbitalen, heliotropen Ödeme bei (paraneoplastischer) Dermatomyositis. (a) Isolierte Lidödeme mit nur sehr diskreter Rötung. (b) Heliotropes Erythem mit Lidödemen und für die Dermatomyositis charakteristischer „perioraler Blässe“. (c) Ausgedehntes heliotropes Erythem mit Lidödemen. (d) Kissenartige Ödeme der Ober‐ und Unterlider sowie Schwellung des gesamten Gesichtes.

Im Zeitraum von 2015 bis 2024 wurden insgesamt 36 Patienten mit gesicherter DM an den Hautkliniken in Oberhausen und Duisburg behandelt. Bei allen Patienten war mindestens einer der in der aktuellen AWMF S2k‐Leitlinie zu Myositissyndromen (https://register.awmf.org/assets/guidelines/030‐054l_S2k_Myositissyndrome_2024‐10‐verlaengert.pdf) aufgeführten Myositis‐spezifischen und/oder Myositis‐assoziierten Autoantikörper nachweisbar. Davon waren 13 (36,1 %) Patienten TIF1‐γ‐positiv und 4 (11,1 %) Patienten NXP‐2‐positiv. Es erfolgte retrospektiv die Datenauswertung von 10 (27,8 %) Fällen mit PHEÖ. Die klinischen und serologischen Charakteristika der Patienten sind in Tabelle 1 zusammengefasst. Das klinische Bild des PHEÖ war primär durch periokuläre Lidödeme gekennzeichnet, die zusammen mit heliotropem Erythem unterschiedlich starker Ausprägung auftraten (Abbildung 1). Von den 10 Patienten waren 7 weiblich und 3 männlich. Das Alter bei Erstdiagnose der DM variierte zwischen 25 und 77 Jahren. Insgesamt konnte bei 9 der 10 Patienten ein Malignom histologisch gesichert werden: Urothelkarzinom (n = 1), maligner neuroendokriner Tumor der Appendix (n = 1), Ovarialkarzinom (n = 2), Pankreaskarzinom (n = 1) sowie nicht‐kleinzelliges Bronchialkarzinom (NSCLC; n = 4). In vier Fällen wurden die Tumoren 1–6 Monate nach der Erstdiagnose der DM gesichert.

**TABELLE 1:** Klinische und serologische Charakteristika der Patienten mit Dermatomyositis und periorbitalem Erythem und Ödem

P	ALTER BEI ED	G	ANA	NACHWEIS RO (SSA)‐AK	MYOSITIS‐AK	RASCHER KRANKHEITS‐BEGINN (< 4 WO)	KUTANE NEKROSEN	CK	DYS‐PHAGIE	ANZAHL PET‐CT	NEOPLASIE	ZEITPUNKT DIAGNOSE‐STELLUNG DER NEOPLASIE	THERAPIE	VERLAUF
1	69	w	‐	‐	TIF1‐γ, PL‐7, Ku	nein	nein	↑	nein	1	NSCLC	2 Monate nach ED DM	GKS, AZA IVIG	lebt, DM gebessert
2	46	w	1:80	‐	TIF1‐γ	ja	nein	‐	ja	5	Vaginales Uptake, bisher kein Malignom gesichert	‐	GKS, AZA IVIG, MTX HCQ, RTX	Progress der DM
3	64	m	‐	‐	TIF1‐γ, MI‐2	ja	nein	↑↑	nein	1	Urothelkarzinom	4 Monate nach ED DM	GKS, AZA IVIG	lebt, DM gebessert
4	44	w	1:5120	positiv	NXP‐2	ja	nein	↑	nein	1	Ovarialkarzinom	6 Monate nach ED DM	GKS, MTX IVIG, HCQ, RTX	lebt, DM fast komplett abgeheilt
5	25	w	1:160	‐	MDA‐5	nein	ja	↑	nein	1*	Neuroendokriner Tumor der Appendix	7 Jahre vor ED DM	GKS, HCQ MTX	lost to follow‐up
6	51	w	1:640	positiv	TIF1‐γ	ja	nein	↑↑↑↑	ja	1	Ovarialkarzinom	1 Mo nach ED DM	GKS	lebt, DM unverändert
7	77	m	‐	‐	TIF1‐γ	ja	nein	↑↑↑	nein	0	NSCLC	bei ED DM		verstorben
8	72	m	1:320	positiv	TIF1‐γ	ja	nein	↑	nein	0	NSCLC	bei ED DM		verstorben
9	75	w	‐	‐	TIF1‐γ	ja	nein	↑↑↑↑↑	ja	0	Pankreaskarzinom	bei ED DM		verstorben
10	59	w	‐	‐	TIF1‐γ	ja	nein	↑	nein	0	NSCLC	bei ED DM	GKS, HCQ	lebt, DM gebessert

*Abk*.: P, Patienten; G, Geschlecht; AK, Antikörper; ANA, Antinukleäre Antikörper; ED, Erstdiagnose; DM, Dermatomyositis; ‐, negativ; ↑, erhöht; ↑↑, circa 3‐fache Erhöhung des Normwertes (bis 145 U/l); ↑↑↑, circa 5‐fache Erhöhung des Normwertes (bis 145 U/l); ↑↑↑↑, circa 10‐fache Erhöhung des Normwertes (bis 145 U/l); CK, Creatinkinase; NSCLC, Nichtkleinzelliges Bronchialkarzinom; GKS, Glukokortikosteroide; AZA, Azathioprin; IVIG, Intravenöse Immunglobuline; RTX, Rituximab; HCQ, Hydroxychloroquin; MTX, Methotrexat; TIF1‐γ, *Transcription‐Intermediary‐Factor‐1 gamma*; PL‐7, Anti‐threonyl‐tRNA‐Synthetase‐7; MI‐2, Anti‐Mi‐2 (Chromodomain‐Helicase‐DNA‐Binding‐Protein‐Komplex); MDA‐5, *Melanoma‐Differentiation‐Associated protein‐5*; NXP‐2, *Nuclear‐Matrix‐Protein‐2*; Ro (SSA), Anti‐Ro; KU, Anti‐Ku (*DNA‐end‐binding protein*‐Komplex). *Das PET‐CT erfolgte im Rahmen der Erstdiagnose DM.

Bei einer Patientin mit Ovarialkarzinom und NXP‐2‐positiver DM (Patientin 4) bildeten sich nach vollständiger operativer Entfernung des Tumors alle Symptome der DM vollständig zurück, bei einem Patienten mit TIF1‐γ‐positivem Urothelkarzinom (Patient 3) kam es postoperativ zu einer deutlichen, klinischen Besserung. Zwei Patienten mit metastasiertem NSCLC (Patient 1 und 10) erhalten aktuell eine Chemo‐Immuntherapie und Radiatio und zeigen eine klinische Besserung der DM. Beide Patienten waren TIF1‑γ‐positiv. Bei Patientin 10 ergab die Basisdiagnostik mittels kontrastmittelgestützter CT des Thorax bereits einen hochgradigen Verdacht auf ein Bronchialkarzinom, woraufhin zeitnah eine histopathologische Sicherung durch eine endobronchiale Ultraschall‐gestützte Biopsie erfolgte. Zwei männliche Patienten im Alter von 77 und 72 Jahren (Patient 7 und 8) sowie eine 75‐jährige weibliche Patientin (Patientin 9) verstarben jeweils kurz nach Diagnosestellung infolge tumorassoziierter bzw. kardiopulmonaler Komplikationen. Bei Patient 7 zeigte die initiale Computertomografie (CT) des Thorax einen spikulierten Rundherd im linken Lungenunterlappen. Aufgrund der peripheren Lage der Raumforderung wurde eine CT‐gestützte perkutane Nadelbiopsie zur histopathologischen Sicherung durchgeführt. Zusätzlich fanden sich im Serum Anti‐TIF1‐γ‐Antikörper sowie eine erhöhte Neuronen‐spezifische Enolase. Dieser Patient verstarb an einer paraneoplastischen Lungenembolie. Bei Patient 8 wurde ebenfalls eine pulmonale Raumforderung identifiziert und als NSCLC gesichert. Auch dieser Patient war TIF1‐γ‐positiv und verstarb zeitnah nach Diagnosestellung infolge des Tumorleidens. Patientin 9 mit TIF1‐γ‐positiver DM verstarb an einem histologisch gesicherten Pankreaskarzinom. Bei initial unklarem Befund im Bereich des Pankreas wurde ergänzend eine Magnetresonanz‐Cholangiopankreatikographie durchgeführt, gefolgt von einer endosonographischen Feinnadelpunktion zur histologischen Sicherung. Bei Patientin 5, bei der bereits sieben Jahre vor der DM‐Erstdiagnose ein maligner neuroendokriner Tumor der Appendix diagnostiziert worden war, zeigte die Positronen‐Emissions‐Tomographie (PET)‐CT zum Zeitpunkt der Erstdiagnose der DM pathologische Anreicherungen in den zervikalen, axillären, mediastinalen und hilären Lymphknoten sowie eine gesteigerte Signalintensität im Bereich der linken Adnexe, was auf eine diffuse Metastasierung hinwies. Klinisch bestanden neben dem PHEÖ auch kutane Nekrosen am Metacarpophalangeal (MCP)‐III‐Gelenk der rechten Hand sowie am rechten Ellenbogen. Serologisch zeigte die Patientin eine Positivität für das Melanom‐Differenzierungs‐assoziierte Protein 5 (MDA‐5, CADM‐140). Der weitere Krankheitsverlauf dieser Patientin (Patientin 5) ist unbekannt (*Lost to follow‐up*).

In 8 von 10 Fällen konnte Anti‐TIF1‐γ nachgewiesen werden. In 7 dieser Patienten wurde ein Malignom histopathologisch gesichert (87,5 % PPW). Bei den zwei TIF1‐γ‐negativen Patientinnen konnte NXP‐2 (Patientin 4) und MDA‐5 (Patientin 5) nachgewiesen werden. Nur eine TIF1‐γ‐positive Patientin (Patientin 2) zeigte unter regelmäßigen PET‐CT‐Kontrollen (insgesamt 5 seit Erstdiagnose der DM) bis dato keine sicheren Hinweise auf eine Neoplasie. Hier zeigte sich jedoch eine diffuse vaginale Signalverstärkung ohne CT‐Korrelation bei unauffälliger gynäkologischer Befundung. Auch die letzte PET‐CT‐Kontrolle blieb ohne pathologischen Nachweis, trotz deutlichem klinischem Progress der DM mit ausgeprägter Muskelschwäche, Dysphagie und generalisierter Asthenie.

In der internationalen Literatur wurden prädiktive Faktoren für das Vorliegen einer paraneoplastischen DM beschrieben.^18^ Diese sind in Tabelle 2 zusammen mit dem in dieser Arbeit beschriebenen PHEÖ aufgeführt. Unter Berücksichtigung dieser Faktoren zeigt sich in unserer Fallserie folgendes Profil:

- Bei allen 10 Patienten (100 %) war ein ausgeprägtes PHEÖ vorhanden
- Anti‐TIF1‐γ‐Antikörper fanden sich bei 8 von 10 Patienten (80 %)
- Erhöhte Kreatinkinase (CK)‐Werte zeigten sich bei 9 Patienten (90 %), davon wiesen 4 Patienten (40 %) stark bis sehr stark erhöhte Werte auf
- 9 von 10 Patienten (90 %) waren bei Erstdiagnose der DM älter als 40 Jahre
- 3 Patienten (30 %) waren männlich
- Kutane Nekrosen traten bei einem Patienten (10 %) auf
- Dysphagie bestand bei 3 Patienten (30 %)
- Ein rascher Krankheitsbeginn wurde bei 8 Patienten (80 %) beobachtet, während die übrigen 2 Patienten (20 %) primär eine amyopathische Form der DM mit rein kutanen Manifestationen zeigten

**TABELLE 2:** Klinische Kriterien für den Hinweis auf ein erhöhtes Risiko für paraneoplastische Dermatomyositis.

Kriterien
Alter > 40 Jahre bei Erstdiagnose
Männliches Geschlecht
Rascher Krankheitsbeginn (< 4 Wochen)
Heliotropes Erythem oder PHEÖ
Dysphagie
Hautulzera oder kutane Nekrosen
CK‐Erhöhung
CRP‐ oder BSG‐Erhöhung
Nachweis von Anti‐TIF1‐γ‐Antikörpern

*Anmerkung*: Diese Kriterienliste ist angelehnt an bisherige Untersuchungen (Lu et al. 2014)^18^ sowie die Charakteristika dieser Fallserie. Sie dient als orientierende Hilfestellung zur Einordnung des Risikos für das Vorliegen einer paraneoplastischen Genese. BSG, Blutsenkungsgeschwindigkeit; CK, Creatinkiase; CRP, C‐reaktives Protein; PHEÖ, Periorbitales heliotropes Erythem und Ödem.

## DISKUSSON

Für die DM sind verschiedene prädiktive Faktoren bekannt, die mit einem erhöhten Risiko für Malignome einhergehen. Eine 2014 veröffentliche Metanalyse konnte für ein erhöhtes Malignom‐Risiko der DM höheres Lebensalter, männliches Geschlecht, kutane Nekrosen, erhöhte Blutsenkungsgeschwindigkeit und C‐Reaktives Protein‐Level sowie Nachweis von Anti‐TIF1‐γ‐Antikörper ermitteln.^18^ Eine weitere Studie zu 192 Patienten mit DM/PM zeigte in der multivariaten Analyse Alter über 40 Jahre (*Odds Ratio* (OR) = 3,44) und heliotropes Erythem (OR = 2,96) als unabhängige Risikofaktoren für Malignom‐Assoziation.^17^ Unseres Wissens nach wurde über das in dieser Arbeit beschriebene PHEÖ und paraneoplastische DM noch nicht berichtet. Eine Übersicht über bisher in der Literatur beschriebene Risikofaktoren für eine paraneoplastische DM ist in Tabelle 2 dargestellt. In unserer Fallserie lag die mittlere Anzahl der jeweiligen prädiktiven Faktoren bei 5 (von maximal 8 möglichen Punkten). Die Kriterien‐Liste soll nicht als diagnostisches Instrument verstanden werden, könnte aber als pragmatische Hilfe bei der Einschätzung des Malignom‐Risikos dienen, um Patienten mit DM zu identifizieren, die sehr kurzfristig einem Malignom‐Screening zugeführt werden sollten.

Der genaue Pathomechanismus des PHEÖ ist weitestgehend unbekannt. Die DM als eine antikörperabhängige und komplementvermittelte Mikroangiopathie führt nicht nur zur perifaszikulären Atrophie, sondern auch zu lokaler Ischämie. C5b‐9‐Komplement‐Komplexe, auch als Membran‐Angriffs‐Komplexe (MAK) bekannt, konnten in Kapillaren und anderen vaskulären Strukturen von Muskel‐ und Hautgewebe nachgewiesen werden.^19^, ^20^ Die mikrovaskuläre Ablagerung des MAK resultiert aus einer klassischen Aktivierung des Komplementsystems, die durch die direkte Bindung von C1q an Endothelzellen initiiert wird. Diese Vorgänge führen zu einer Dysfunktion des Endothels mit konsekutiver Erhöhung der Gefäßpermeabilität, wodurch die Entstehung von Ödemen infolge extravasaler Flüssigkeitsansammlung begünstigt wird. Dabei ist die dünne Haut der periorbitalen Region bei DM besonders vulnerabel. Das charakteristische, heliotrope Exanthem der DM mit rötlich‐violetter Hautverfärbung lässt sich durch lokale Entzündungsprozesse (perivaskuläre Infiltrate und eine Interface‐Dermatitis) sowie durch hämodynamische Stauungsmechanismen infolge einer reduzierten Kapillardichte erklären. Derartige mikrovaskuläre Veränderungen begünstigen eine lokale Ischämie, die ihrerseits hypoxieinduzierte Gewebeveränderungen und eine weitere Erhöhung der Gefäßpermeabilität nach sich zieht. Diese pathophysiologischen Mechanismen verdeutlichen den ätiologischen Zusammenhang zwischen Ödem und Erythem und könnten als Erklärung für die klinische Manifestation von PHEÖ in dieser besonders vulnerablen, anatomischen Region dienen.^19^, ^20^

Mit Ausnahme eines Falls konnte bei allen Patienten im Zusammenhang mit der DM ein Malignom histologisch gesichert werden. In unserem Patientenkollektiv war in 78% (7 von 9 Patienten) der paraneoplastischen DM‐Fälle Anti‐TIF1‐γ nachweisbar. In der Literatur variieren die Angaben hierzu stark und liegen zwischen 14% und 100%.^6^, ^9^, ^12^, ^21^, ^22^ Interessanterweise umfasste unser Kollektiv eine Anti‐TIF1‐γ‐positive Patientin mit schwerer DM, gekennzeichnet durch persistierendes, ausgeprägtes PHEÖ sowie fortschreitende Muskelschwäche und Muskelatrophie, bislang jedoch ohne sichere Malignom‐Assoziation. Trotz multipler Therapieansätze (systemische Glukokortikosteroide, Azathioprin, Hydroxychloroquin, Methotrexat, Mycophenolat‐Sodium, intravenöse Immunglobuline sowie Rituximab) zeigte sich bislang keine wegweisende, klinische Besserung. Auffällig war zudem das durchgehend normale Kreatinkinase‐Profil, was die Diagnose einer amyopathischen DM bei dieser Patientin unterstützt. Andere Myositis‐spezifische Antikörper wie MDA‐5 und NXP‐2 konnten nur bei 2 Patienten detektiert werden. Patienten mit Anti‐NXP‐2‐Antikörper zeigen häufig eine ausgeprägte Muskelbeteiligung mit schweren Myalgien, Dysphagie sowie Schwäche der proximalen und distalen Muskulatur. Darüber hinaus ist auch für Anti‐NXP‐2‐positive Patienten ein bis zu 3,7‐fach erhöhtes Risiko für Malignome im Vergleich zur erwarteten Prävalenz in der Allgemeinbevölkerung beschrieben worden.^23^

Zum Zeitpunkt der Erstvorstellung wiesen alle Patienten des untersuchten Kollektivs ein PHEÖ in unterschiedlicher Schwere und Ausprägung auf, das von isolierten Lidödemen bis zu kompletter Gesichtsschwellung reichte. Kürzlich wurde über das Auftreten von unilateralen heliotropen Erythemen (und Ödemen) berichtet, wobei nur eine Orbitaregion betroffen war.^24^ Bei den 13 bisher beschriebenen derartigen Fällen in der Literatur konnten im Gegensatz zu unserer, überwiegend TIF1‐γ‐positiven Fallserie, MDA‐5‐Autoantikörper detektiert werden; zudem lag bei den Patienten häufig auch eine interstitielle Lungenerkrankung vor.^24^

Die Einordnung des PHEÖ als prädiktiver Hinweis auf eine Malignom‐Assoziation bei DM, insbesondere im Vergleich zu den zuvor bereits beschriebenen Risikofaktoren, erfordert größere Fallzahlen, die im Rahmen retrospektiver Analysen oder Metaanalysen mit gezielter Erfassung dieses klinischen Merkmals oder idealerweise durch prospektive Studien erhoben werden sollten. Unsere Beobachtungen unterstreichen die Relevanz klinischer und serologischer Marker für die frühzeitige Erkennung einer potenziell paraneoplastischen DM, wie dies in der aktuellen AWMF S2k Leitlinie für Myositissyndrome beschrieben wird. Besonders die Kombination aus serologischem Risikoprofil (Anti‐TIF1‐γ‐ oder Anti‐NXP‐2‐Positivität) und charakteristischen, klinischen Leitsymptomen wie dem hier beschriebenen PHEÖ sollte als Hinweis auf eine potenziell paraneoplastische DM verstanden werden und Anlass zu einer zügigen und umfassenden Tumorsuche geben.

## DANKSAGUNG

Open access funding enabled and organized by Projekt DEAL.

## INTERESSENKONFLIKT

Keiner.
